# Provings and Clinical Observations with High Potencies

**Published:** 1892-11

**Authors:** Malcolm Macfarlan

**Affiliations:** Philadelphia


### PROVINGS AND CLINICAL OBSERVATIONS WITH HIGH POTENCIES.

MALCOLM MACFARLAN, M. D., PHILADELPHIA.

### III—Repertory.

### SIDES, GENERAL SYMPTOMS BACK AND NECK.

ERECTHITES $^{10M}$.

Pain and soreness across the small of the back.

(SAPO-CAST) 10th deçimal.

Frequent sensations commenced like an electric shock from small of back, ran up the spine to the head and thence to epigastrium; chilly in the back with tired and sore feeling there.

### Arsenic $^{6M}$.

Dorsum painful ; soreness and pain across buttocks, extending into head and right shoulder, and whole right side feels as if swollen.

Fainty ; great weakness ; giddy ; dizzy. Compelled to lie down frequently, so weak.

### Plantago-minor $^{11C}$.

Burning pain across the buttocks, often verified.

### Muriatic-acid $^{5C}$.

Wants to lie abed most of the day, so tired.

### Woorari $^{90M}$.

Her lips appear to get blue, accompanied with fever, which comes and goes.

### Sulph-ac.$^{5C}$.

Feels tired and exhausted ; soreness between the scapulæ.

### Sapo-soda $^{2C}$.

All her joints ache ; stiff and aching from head to foot.

### Wisteria $^{5C}$.

Sudden and severe sharp pain across the small of the back on both sides of it ; never had this before, couldn't stoop, pain would seize her so quickly.

### Psorinum $^{42M}$.

Severe pain at the right side opposite tenth rib.

### Senega$^{5C}$.

She has to press in at her left side over tenth rib to find relief it hurts her so ; gnawing pain relieved by deep, continued pressure.

### Sanguinaria$^{24M}$.

Soreness down the muscles of his back ; feels it mostly on breathing ; pain shifts about.

### TEREBINTH.$^{17M}$.

Sharp pain in small of the back ; lumbar region.

### ADEPS $^{1M}$.

Acute pain between last bone of lumbar vertebræ and sacrum.

### AGARICUS-MUSC.$^{47M}$.

General muscular stiffness ; back of neck mostly affected.

### APOCYNUM-CANN.$^{6M}$.

General stiffness of the legs and body ; bends his body with great difficulty.

### ARANEA-DIAD.$^{45M}$.

Her back is so painful she cannot sleep well because of it, hurts her continually.

Felt very tired ; feels as if she would drop after very little exertion.

### ARSENIC $^{6M}$.

Sensation of fainting caused in many provers ; symptoms more marked when out in the air.

### AURUM-MUR.$^{5M}$.

General severe backache.

### BELL.$^{101M}$.

Soreness in region of liver and above the epigastrium.

### BURSA-PASTORIS $^{9M}$.

General bruised feeling throughout the body.

### CANCER-ASTAC $^{5C}$.

Nervous movements all over his body.

### CIMICIFUGA-RAC.$^{95M}$.

Backache mostly in the small of the back, across the loins.

### CORNUS-FLORIDA$^{45M}$.

Very severe aching pain on both sides of the waist line.

Pains run up the left side of the trunk like the shock from a battery; pains cause violent contraction of the body.

Cramps from the side of the waist extending toward the ovary.

### COCHLEARIA-ARMORA$^{10M}$.

Severe contractive sensation in left iliac region.

### CUPRUM-ACET.$^{45M}$.

Sensation on the fourth day as if the top of her sacrum was injured; backache referred to upper part of the sacrum in several provers.

### DIGITALIS-PURP.$^{CM}$.

Produced throbbing in every part of the body, aggravated by pressure; frequent painless watery stools.

### ERECHTHITES-HIERA$^{10M}$.

Stitches in the middle of the back; cold feelings in back and legs.

### EUPATORIUM-PERFOL.$^{CM}$.

Pains all over him, somewhat similar to muscular rheumatism.

### FORMICA-RUFA$^{45M}$.

Tired feeling referred to his back; felt slightly giddy.

### HYPERICUM-PERF.$^{45M}$.

Flesh sore and bruised all over the body. This symptom occurred in all the provers, and was not long lasting.

### KALI-CARB.$^{24M}$.

Violent pain in the *small of the back;* wants the back pressed, because it brings relief; bearing-down sensation in the pelvis.

### LACH.$^{CM}$.

Rolling from side to side in bed, can't stop it; complete or uncontrollable restlessness; desire to be in the open air; sensation of constriction about the throat.

### Linaria-vulg.$^{2C}$.

Fainty feeling three or four times daily ; *fainted completely away* once. This never happened before, prover was however weakly. Produced *fainting* in another case ; man otherwise in fair health, no other severe symptom ; free and frequent urination.

### Merc.-viv.$^{101M}$.

Sore, bruised sensation all over the body ; pains shoot about in various parts ; can't bear the least noise ; loins pain him ; aching from head to foot ; left side of throat sore, tonsil sensitive.

### Jaspis$^{2C}$.

Aching in region of kidneys ; slight dyspeptic symptoms ; eructations ; distress at the epigastrium.

### Merc.-iod.$^{2C}$.

Feels as if generally bruised ; dryness of throat with soreness ; constant disposition to swallow ; ptyalism.

### Arsenic$^{6M}$.

As soon as he makes an effort to move, he feels very weak ; when lying down this passes away ; nausea ; sleeplessness and anxiety.

### Nitric-acid$^{5C}$.

Soreness across both buttocks.

### Phosphorus$^{CM}$.

Nervous, excitable ; backache ; burning sensation in the throat and stomach ; pains in the right side of the small of the back, mostly.

### Phytolacca-decan.$^{47M}$.

Pain and soreness across kidneys, felt mostly on deep pressure, and extending to buttocks as well ; dull pain across forehead ; can't stand in morning ; feels wretched on getting up ; aching from shoulders to hips.

### Plantago-min.$^{11C}$.

Pain in his back; severe burning pain across her buttocks; thinks she ought to raise herself out of bed to be relieved; twitching of the muscles of the extremities generally.

### Ratanhia.

Feels very weak; confused mentally, even while doing simplest things.

### Uva-ursi $^{10M}$.

Slight symptoms of cold in the head; general muscular soreness as if bruised; soreness in the chest walls.

### Merc-corr.$^{5C}$.

Feeling of exhaustion; left side mostly affected; has not strength to work; soreness at the waist; left side mostly.

### Rhus-tox.$^{105M}$.

Caused rheumatic pains; produced such intolerable itching all over the body that it was nearly unbearable; constantly rubbing and scratching to relieve the general itching of the skin; skin reddens on the least rubbing, and disposed to raise in welts.

### Sambucus-nig.$^{45M}$.

Soreness and aching in the muscles generally.

### Sarracenia-purp.$^{2M}$.

Feeling of great weakness and soreness between the shoulders and below them.

### Strontia-carb.$^{5C}$.

Chilly, creepy sensations about the small of her back, which is slightly sensitive and painful.

### Merc-vir.$^{101M}$.

Aching in the lower extremities, at the waist and small of the back.

